# Endpoints in Dysphagia Trials. Comment on Speyer et al. Neurostimulation in People with Oropharyngeal Dysphagia: A Systematic Review and Meta-Analyses of Randomised Controlled Trials—Part I: Pharyngeal and Neuromuscular Electrical Stimulation. *J. Clin. Med*. 2022, *11*, 776

**DOI:** 10.3390/jcm11123302

**Published:** 2022-06-09

**Authors:** Rainer Dziewas, Philip M. Bath

**Affiliations:** 1 Department of Neurology and Neurorehabilitation, Klinikum Osnabrück—Academic Teaching Hospital of the WWU Münster, Am Finkenhügel 1, 49076 Osnabrück, Germany; 2 Stroke Trials Unit, Mental Health & Clinical Neuroscience, University of Nottingham, Nottingham NG5 1PB, UK; philip.bath@nottingham.ac.uk

We read with interest the authors’ systematic review and meta-analysis of pharyngeal electrical stimulation (PES) and neuromuscular electrical stimulation (NMES) in patients with oropharyngeal dysphagia (OD) [1]. Neurostimulation techniques are increasingly used in the rehabilitation of dysphagia and clearly constitute an emerging field of modern medicine. Recently, several meta-analyses have been dedicated to this topic, which is also covered in current guidelines [2,3]. As observed by Speyer and co-workers, most of these meta-analyses have focused on specific disease categories, which was the reason why the authors aimed for a wider approach that included any medical diagnoses. While we appreciate this intention and agree that this meta-analysis adds to current knowledge, we feel that the authors missed their self-set goal of providing the full picture regarding the treatment effects of PES in four respects.

First, the authors excluded two of eight identified randomised controlled trials of PES because they assumed, as stated in the results section, that there was “no confirmation of OD diagnosis prior to treatment” in these studies [4,5].

In our opinion, the exclusion of these studies based on the reasoning put forward by the authors is not warranted. The two studies in question, a single-centre pilot study [4] and a multicentre trial [5], applied PES in tracheotomised stroke patients who had been weaned from mechanical ventilation but could not be decannulated because of severe dysphagia, a rather common situation in neurocritical care. Both studies used a well-established FEES-based protocol [6,7,8] to assess airway safety, a key and fundamental aspect of swallowing function, before and after intervention, i.e., PES or sham. This protocol consists of a stepwise evaluation of the following three items:

(i) Assessment of secretion: Does the patient present with a massive pooling of saliva or a silent penetration/aspiration of saliva?
(ii) Assessment of spontaneous swallows: Does the patient present with <1 swallow per minute and/or a missing “whiteout”?
(iii) Assessment of laryngeal sensibility/cough: Does the patient have anaesthesia and/or no effective cough upon gently touching the aryepiglottic region with the tip of the endoscope?

If all three items are rated as passed, the patient is considered ready for decannulation; if the patient fails at one or more steps, decannulation needs to be postponed. While this protocol has specifically been developed for examining tracheotomized patients, all of its items are part of the standard FEES protocol, as recently summarised in an FEES tutorial jointly issued by of the American Speech–Language–Hearing Association and by the American Board of Swallowing and Swallowing Disorders [9], and have been found to be of clinical relevance in different areas of dysphagia management.

Hence, we feel that it is inappropriate to exclude the two mentioned PES trials from the meta-analysis. PES was associated with a more than 10-times greater chance of treatment success as compared to sham in these trials, so their inclusion in the meta-analysis would have most likely impacted on the estimated treatment effect of PES.

Second, the authors focused on the effect of PES on penetration aspiration scale score rather than relevant clinical measures such as the dysphagia severity rating scale [10]. A previous meta-analysis found that PES improved DSRS [11].

Third, the authors suggested that “NMES may have more promising effects compared to PES” [1]. We are unaware of any head-to-head comparisons of the two techniques (and none were presented in the systematic review), so it is inappropriate to comment on the superiority of one technique over the other based on the indirect comparison of the meta-analysis results. For example, the between-group lower and upper confidence limits of effect overlapped (NMES 0.105 to 0.760; PES −0.170 to 0.368) which is compatible with no difference in efficacy.

Lastly, the introduction of interventions into routine clinical practice requires more than just evidence of efficacy; we also need evidence that interventions are cost-effective, and this information is missing from this and other meta-analyses.

In conclusion, it is likely that both techniques are effective and have a clinical role in overlapping groups of patients. However, as the authors say, large trials are needed, and these should include both clinical and health economic outcome measures. We are delighted to announce that the UK government-funded Pharyngeal Electrical Stimulation for Acute Stroke Dysphagia Trial (PhEAST) is studying the clinical and cost effectiveness of PES versus control in 800 patients with dysphagia after a recent stroke (https://stroke.nottingham.ac.uk/pheast/, accessed 4 March 2022).
